# Analysis of Oxidative Stress-Related Markers in Crohn’s Disease Patients at Surgery and Correlations with Clinical Findings

**DOI:** 10.3390/antiox8090378

**Published:** 2019-09-06

**Authors:** Cristina Luceri, Elisabetta Bigagli, Sara Agostiniani, Francesco Giudici, Daniela Zambonin, Stefano Scaringi, Ferdinando Ficari, Maura Lodovici, Cecilia Malentacchi

**Affiliations:** 1 Department of Neurosciences, Psychology, Drug Research and Child Health (NEUROFARBA), Section of Pharmacology and Toxicology, University of Florence, 50139 Florence, Italy; 2 Department of Experimental and Clinical Medicine, University of Florence, 50134 Florence, Italy; 3 Department of Experimental and Clinical Medicine, Surgery Unit IBD, Careggi-University Hospital (AOUC), 50139 Florence, Italy; 4 Department of Biomedical Experimental and Clinical Sciences “Mario Serio”, University of Florence, 50134 Florence, Italy

**Keywords:** Crohn’ disease, biomarkers, oxidative stress

## Abstract

Crohn’ disease (CD) patients are at high risk of postoperative recurrence and new tools for the assessment of disease activity are needed to prevent long-term complications. In these patients, the over-production of ROS generated by inflamed bowel tissue and inflammatory cells activates a pathogenic cascade that further exacerbates inflammation and leads to increased oxidative damage to DNA, proteins, and lipids. We measured the products of protein/lipid oxidation and the total antioxidant capacity (ferric reducing ability of plasma, FRAP) in the serum of CD patients with severe disease activity requiring surgery with the aim to characterize their redox status and identify associations between oxidative stress-related markers and their clinical characteristics. At the systemic level, CD was associated with increased levels of protein and lipid oxidation products when compared to healthy volunteers, even though the FRAP values were similar. Advanced oxidation protein product (AOPP) levels showed the highest difference between patients and the controls (11.25, 5.02–15.15, vs. 1.36, 0.75–2.70, median, interquartile range; *p* < 0.0001) and the analysis of receiver operating characteristic (ROC) curves, indicated for AOPP, the best area under the curve (AUC) value for CD prediction. Advanced glycated end-products (AGEs) were also significantly higher in CD patients (*p* < 0.01), which is of interest since AOPP and AGEs are both able to activate the membrane receptor for advanced glycation end products (RAGE) involved in inflammatory diseases. Thiobarbituric acid reactive substance (TBARS) levels were significantly higher in CD patients with ileal localization and aggressive disease behavior, in smokers, and in patients suffering from allergies. In conclusion, our data indicate that circulating oxidative stress biomarkers may be attractive candidates as disease predictors as well as for clinical or therapeutic monitoring of CD. Our results also suggest that AOPP/AGEs and RAGE signaling may represent a pathogenic factor and a potential therapeutic target in CD.

## 1. Introduction

Crohn’s disease (CD) is a chronic inflammatory disorder of the intestinal tract, with increasing prevalence worldwide [1,2]. It is generally accepted that CD as well as ulcerative colitis (UC) are the result of complex interactions among environmental factors, dysregulated immune system, gut microbiota, and disease susceptibility genes [3]. Accumulating data suggest that oxidative stress is at the crossroad between these multiple mechanisms [4,5,6].

Both chronic inflammation and immune system hyperactivation are accompanied by abnormally high levels of reactive oxygen species (ROS) and decreased antioxidant defenses, resulting in oxidative stress. Oxidative stress leads to mucosal layer damage and bacterial invasion, which in turn further stimulate the immune response and contribute to disease progression [7]. One of the main advantages of oxidative modifications of cellular proteins, lipids, and nucleic acids is that they can be measured not only in the affected intestinal tract, but also at the systemic level; several studies have in fact reported increased levels of oxidative stress biomarkers in the serum/plasma of inflammatory bowel disease (IBD) patients [8]. This is of interest at least for two reasons: on one hand, circulating biomarkers of oxidative stress offer the advantage of easy collection, low costs, and the possibility to be used on a large scale; on the other hand, the systemic oxidative stress observed in CD may likely contribute to the development of extra-intestinal manifestations such as perianal fistulas, dermatologic diseases, and arthritis, which are very common in these patients [9].

Circulating antioxidant capacity also seems to be correlated with the clinical status of the patients. Plasma free thiols were recently reported to be associated with favorable outcome in CD, being negatively correlated with biomarkers of inflammation [10], and serum free thiols and uric acid were significantly lower in active CD patients with anemia [11]. Furthermore, a very strong positive correlation was found between the endoscopic activity index and the serum total oxidant status in CD patients under regular follow-up [12]. These data suggest that the measure of circulating oxidative stress markers might be clinically useful both for early diagnosis as well for clinical monitoring. Clinical diagnosis of CD can be complex and it is often delayed. Moreover, CD patients need an adequate assessment of disease activity either to guide clinical treatment, prevent long-term complications, or induce a long-term remission after surgery.

On these bases, the aim of the present study was to explore the association between several peripheral biomarkers of oxidative stress and the clinical characteristics of a cohort of CD patients characterized by therapeutic failure and a complicated disease requiring surgery.

## 2. Methods

### 2.1. Study Population

The study protocol was approved by the Ethical Review Committee of the Hospital of Careggi, Florence, Italy. Written informed consent was obtained from all eligible participants. A total of 71 subjects (54 patients with CD and 17 controls) were included in this observational study. Patients were recruited between January 2015 to January 2017 at the Digestive Surgery Unit of the Careggi Hospital, where all had severe relapse (CD activity index scores of >200) requiring surgery. The healthy volunteers were recruited among the personnel of the Careggi Hospital. Serum was obtained from blood samples, taken at surgery for CD patients or at enrolment for the control subjects, collected in Vacutainer ® collection tubes, coagulated at room temperature, and centrifuged at 1800× *g* for 10 min before the distribution of the supernatant in cryo-tubes, and stored at −20 °C until analysis.

Information on gender, age, disease duration, diagnostic delay, smoking habits, location, disease behavior, extra-intestinal manifestation, perianal disease, recurrence, number of operations, immunological comorbidity, familiarity IBD, and therapy were collected. Demographic and clinical characteristics of CD patients and healthy controls are reported in Table 1.

### 2.2. Ethics Approval and Consent to Participate

This study was approved by the Ethical Committee of Careggi-University Hospital (AOUC), Florence, Italy, on May 2, 2011, protocol no. 2011/0016888, rif. 95/10, authorization Gen Dir 17/572011 protocol no. 2011/0018055, and written informed consent was obtained from all study subjects.

### 2.3. Ferric Reducing Activity of Plasma (FRAP)

A FRAP reagent solution was freshly prepared by mixing 300 mM acetate buffer, pH 3.6, TPTZ solution (10 mM 2,4,6-tripyridyl-s-triazine (TPTZ) in 40 mM HCl), and 20 mM FeCl3·6H_2_O in a volume ratio of 10:1:1. To perform the assay, 0.9 mL of FRAP reagent, 90 μL of distilled water, and 30 μL of serum were mixed and incubated at 37 °C for 30 min. The absorbance was measured at 595 nm. The antioxidant potential of samples was determined from a standard curve plotted using the FeSO4·7H_2_O [13].

### 2.4. Advanced Oxidation Protein Product (AOPP)

For AOPP determination, 20 µL of serum and 980 µL of PBS were mixed to 50 µL of KI 1.16 M and 100 µL of acetic acid. The absorbance of the reaction mixture was immediately read at 340 nm. AOPP were quantified in µmol/mg of proteins using Chloramine-T (Sigma-Aldrich, Milan, Italy) as the standard for the calibration curve [14].

### 2.5. Carbonyl Residues (CO)

Carbonyl residues were determined following the method of Correa-Salde and Albesa [15] with a few modifications. Serum samples (100 µL) were treated for 1 h at room temperature with 900 µL of 0.1% dinitrophenylhydrazine in 2 M HCl and precipitated with 400 µL of 10% trichloroacetic acid (TCA) before being centrifuged for 20 min at 4 °C at 10,000× *g*. The pellets were extracted with 500 µL of ethanol:ethyl acetate mixture (1:1) and centrifuged for 3 min at 4 °C at 10,000× *g*, three times and then dissolved in 15 mL of 6 M guanidine HCl in 20 mM potassium phosphate buffer (PBS), pH 7.5. The solutions were incubated at 37 °C for 30 min and insoluble debris was removed by centrifugation. The absorbance was measured at 370 nm.

Carbonyl content was calculated using a molar absorption coefficient of 22,000 M^−1^ cm^−1^ and expressed as nmol/mg of proteins. Protein content was estimated by using the Bio-Rad DC protein assay kit (Bio-Rad, Segrate, Milan, Italy).

### 2.6. Thiobarbituric Acid Reactive Substances (TBARS)

TBARS were evaluated as an index of lipid peroxidation according the method by Dietrich-Muszalska et al. [16]. A total of 100 µL of serum was first deproteinized by adding 100 µL of TCA, then 160 µL was added to 32 µL of 0.12 M thiobarbituric acid (Sigma-Aldrich, Milan, Italy) in TRIS 0.26 M, and heated for 15 min at 100 °C. The reaction was stopped by placing the vials in an ice bath for 10 min and after centrifugation (at 1600× *g* at 4 °C for 10 min), the absorbance of the supernatant was measured at 532 nm (Perkin Elmer Wallac 1420 Victor3 Multilabel Counter).

TBARS content was calculated using a molar absorption coefficient of 1.56 × 10^−5^ M^−1^ cm^−1^ and expressed as µM.

### 2.7. Advanced Glycated End-Products (AGEs)

AGEs were determined by exploiting the characteristic autofluorescence of the large part of AGEs as described by Cournot and Burillo [17]. A total of 100 µL of 1:5 diluted in PBS serum, were placed in a 96-well plate and the fluorescence intensity was read at 460 nm, after excitation at 355 nm. Results were expressed as arbitrary units (AU).

### 2.8. Statistical Analyses

Statistical analyses were performed using Statgraphics Centurion XVI software and Graph-Pad Prism 7.00. *p*-values less than 0.05 were considered statistically significant. Normality was verified with the Kolmogorov–Smirnov test. Normally distributed and continuous variables were expressed as means ± standard deviation (SD). Non-normally distributed variables were expressed as median and interquartile range. Comparison of continuous variables between the two groups were performed using the Student’s t-test (normally distributed) or Mann–Whitney test (non-normally distributed). Differences between proportions were assessed using the chi-square or Fisher exact test.

The area-under-curve (AUC) of the receiver operating characteristic (ROC) curves for each oxidative stress biomarker were used to characterize their utility for discriminating CD patients from healthy subjects.

A stepwise multiple linear regression analysis with backward selection was performed with oxidative stress markers as the dependent variables and the following factors as independent variables: age at surgery, gender, diagnostic delay, smoke habit, CDAI, disease location, disease behavior, disease duration, extra-intestinal disease, perianal disease, first clinical presentation, recurrence, number of surgeries, allergies, and family history of IBD.

An oxidative score was calculated and consisted of four components. For oxidative markers (TBARS, CO, AGEs, and AOPP) values below the median value were assigned 0 point and those above the median value, 1 point. For FRAP values, the point assignment was the reverse (0 below the median and −1 above the median value). According to the sum of the four components, patients were allocated to three oxidative score categories: low, medium, and high.

## 3. Results

### 3.1. Baseline Characteristics

No significant differences in age, gender, and smoke habit distribution were observed between CD patients and the control group.

At the time of surgery for CD, 16 patients were smokers, 15 former smokers, and 19 had never smoked. In about 54% of the patients, the disease was localized in the ileum, in 34.6% in the colon and only 6 (11.5%) patients had a disease involving both segments. Many of the patients (41%) presented extra-intestinal diseases (skin and arthritis), 45.10% perianal disease, 55.5% had already undergone surgery, and in 30.6% of the cases, familial IBD was observed.

### 3.2. Oxidative Damage and Antioxidant Capacity

**Figure 1:**
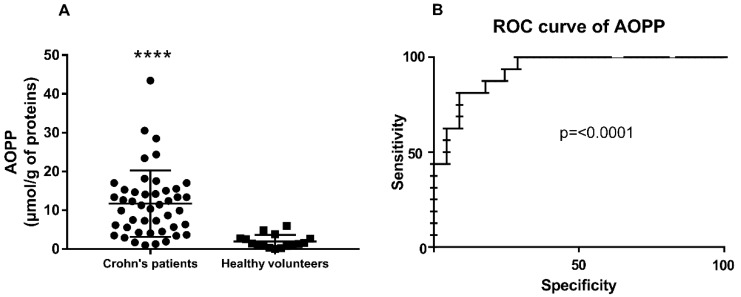
(**A**) Scatter dot plot of the advanced oxidation protein products (AOPP) levels in the serum of Crohn’ patients and healthy volunteers. **** *p* < 0.0001 by Mann–Whitney test. (**B**) ROC curve for AOPP.

All of the oxidative damage parameters measured were significantly higher in the serum of CD patients when compared to the controls (Table 2). In particular, the difference between the AOPP levels in the serum of CD patients was very high when compared to healthy volunteers with a median value of 11.25 (5.02–15.15) vs. 1.36 (0.75–2.70) µmol/g of proteins, respectively (Table 2 and Figure 1A).

Based on the analysis of ROC curves, we assessed the diagnostic utility of the oxidative damage biomarkers as predictors of CD. The area under the ROC curve (AUC) was 0.6938 for TBARS, 0.7412 for CO, 0.7195 for AGEs, and 0.5765 for FRAP. The AOPP determination resulted in a much higher AUC value of 0.9306 for the prediction of Crohn’s disease (Figure 1B).

The antioxidant capacity, measured as FRAP values, in the CD patients and controls was similar (Table 2).

Through univariate analysis, we observed that CD patients treated with azathioprine (*n* = 24) presented FRAP levels significantly higher (*p* < 0.05) than those untreated (*n* = 18). Moreover, serum AOPP levels were significantly reduced in patients treated with mesalazine (*p* < 0.01), but the number of untreated patients was very low (6 vs. 41).

**Figure 2:**
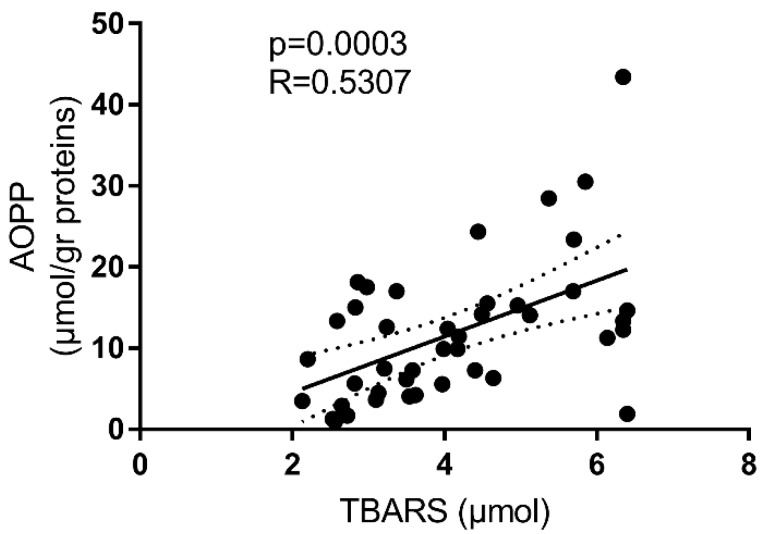
Correlation between AOPP and TBARS levels in the serum of Crohn’s patients.

Positive correlations existed among almost all of the different markers of oxidative stress, both in the CD patients and in the controls (Tables S1 and S2). Figure 2 shows the correlation between circulating AOPP and TBARS levels in CD patients. No correlation between CDAI and oxidative-stress parameters was observed.

### 3.3. Multiple Regression Analysis

Multiple regression analysis identified five independent variables associated with circulating TBARS in CD patients; in contrast, the other oxidative stress biomarkers did not show significant associations with clinical parameters. By backward stepwise regression, TBARS were associated to the disease site, behavior, and first clinical presentation, being higher in patients with an ileal localization of the disease, with a fistulizing and stricturing behavior and with the severity of the first clinical presentation (combination of occlusion, anemia, and weight loss or the presence of perianal fistulas). Moreover, TBARS were also associated with the smoke habit and with the presence of allergies (Table 3). The oxidative score was associated with smoke habit (*p* = 0.013) and with the presence of a skin extension of the disease (*p* = 0.0245) (Table 4).

## 4. Discussion

Accumulating evidence indicates that oxidative stress is not only merely a consequence of chronic inflammation, but may have an essential role in the development and maintenance of inflammation and aberrant immune response in CD. In this regard, our results demonstrate an overall increase in oxidative stress biomarkers in CD patients at surgery when compared to the controls, highlighting that severe clinical activity is reflected by systemic oxidative stress. Among the markers analyzed, AOPP demonstrated the greatest diagnostic ability in differentiating CD patients from the controls. AOPPs are di-tyrosine-containing and cross-linking products, formed by the reaction of plasma proteins, mainly albumin, with chlorinated compounds resulting from the activity of myeloperoxidase (MPO) [14,18]. For this reason, AOPP are recognized as both oxidative protein damage markers and mediators of inflammation.

Increased plasma AOPP levels in patients with chronic diseases including active CD patients have been reported by others [19,20,21]. In our study, CD patients had very high levels of AOPP in their serum, much higher when compared to the study by Krzystek-Korpacka and co-workers [20], who measured a mean level of 1.87 µmol/g of albumin in the plasma of active CD patients. We measured AOPP levels in the serum of CD patients at surgery, therefore with severe clinical conditions, and these high levels can be a consequence of their clinical status.

Interestingly, some mechanistic studies have demonstrated the role of AOPP in the pathogenesis and progression of CD. There is in fact in vitro and in vivo evidence that AOPPs induced depletion of intestinal epithelial cells and inflammatory changes that alter the structural integrity of the intestinal mucosa [22,23,24]. These compounds are also able to modulate cell cycle arrest [25]; Shi and co-worker recently demonstrated that AOPPs exhibit their negative regulatory function on intestinal epithelial cell cycle progression by activating the RAGE/CD36-c-jun N-terminal kinase (JNK)-p27kip1 signaling pathway [21]. By interacting with RAGE and CD36 receptors, AOPP activate protein kinase C and nicotinamide adenine dinucleotide phosphate (NADPH) oxidase as well as the NF-κB-dependent inflammatory pathway [26]. Xu et al. [27] also found that the deposition of AOPPs in fibrotic lesions from CD patients promoted epithelial–mesenchymal transition, a fundamental mechanism in complications of CD such as intestinal fibrosis through the oxidative and inflammatory pathway.

Other than AOPP, the first identified ligands of RAGE were AGEs. Although RAGE/AGE signaling has mainly been studied in diabetes, there is also evidence of its activation in neurodegenerative diseases, cancer, and in various inflammatory diseases including CD [28,29,30]. Mice lacking RAGE receptors are in fact protected from chemically-induced intestinal inflammation and treatment with a RAGE-specific inhibitor protects mice from indomethacin-induced enteritis and dextran sulfate sodium-induced colitis, suggesting that the RAGE signaling pathway could be a promising therapeutic target for IBD patients [30]. On the contrary, there are no reports available in the literature on the serum level of AGEs in patients with CD. AGEs are heterogeneous compounds mainly generated through the non-enzymatic glycation of protein, lipids, and nucleic acid driven by hyperglycemia and oxidative stress, but increased serum levels were associated with hypercholesterolemia [31] and cigarette smoking [32]. In addition, humans are exposed to dietary sources of AGEs through animal-derived foods and cooking processes that result in the formation of new AGEs [33]. Moreover, AGEs such as glycol aldehyde and 2-hydroxy-propanal may be generated by activated neutrophils, even in the absence of sugars [34].

Lipid peroxidation products are also implicated in the pathogenesis of IBD. For instance, it has been reported that 4-hydroxynonenal treatment suppressed colonic expression of tight junction proteins, enhanced bacterial translocation from the gut into the systemic circulation, and increased activation of Toll-like receptor 4 signaling [35].

In our cases, circulating TBARS were significantly higher when compared to the controls and these results are in line with those obtained by others who measured elevated lipid peroxidation markers malondialdehyde (MDA)/TBARS in CD patients [35,36,37,38]. In the study by Sampietro and co-workers, CD patients at surgery showed a significantly higher basal peroxidative state when compared to the controls, but while the inflammatory and oxidative indices were significantly reduced, two months later, and maintained low one year after surgery, TBARS did not reach levels comparable to those in the control subjects, indicating that in quiescent CD, there is an upregulated level of plasma peroxidation [39].

Szczeklik and co-workers recently described the presence of an upward trend in the serum (and saliva) MDA levels, depending on the severity of CD and a correlation between the MDA levels and the visible symptoms of inflammation [40]. Our results showed a significant correlation among lipid peroxidation, disease location, and behavior and with the severity of the first clinical presentation of CD. All these data suggest the role of TBARS as a potential marker of the severity of the disease.

Serum TBARS levels were also correlated with smoking habit being higher in smokers and to a lesser extent, in former smokers when compared to non-smokers. Cigarette smoke is a well-known source of ROS and one of the most powerful oxidative stress inducers. It is considered both an etiological risk factor for CD as well as for its recurrence; smokers have a higher risk when compared to non-smokers of developing a postoperative recurrence and the risk increases in relation to the number of cigarettes smoked [41,42].

We also defined an oxidative score to take into account the overall oxidative–antioxidant status of each patient and identified positive correlations with smoking habit and the presence of cutaneous manifestation of the disease, a well-recognized complication of IBD that frequently occurs in CD and is associated with a worse prognosis [43,44]. The pathogenic mechanism underlying the development of cutaneous manifestations in CD patients is still not known, but our results suggest that oxidative stress may have a role.

Despite the high degree of oxidative stress observed in CD patients, witnessed by the increase in all oxidative markers measured, all correlated to each other, we did not observe differences in their antioxidant status, measured as FRAP, when compared to controls. On the contrary, several studies reported a reduced antioxidant capacity in CD patients, measured both as plasma carotenoids and other vitamin content [45,46], or as total antioxidant capacity [12,47,48], or as plasma free thiols [10]. In particular, Bourgonje and coworkers reported strong and negative correlations among albumin-adjusted plasma free thiols and a number of pro-inflammatory markers of disease activity [10]. In this study, a more favorable redox status was also observed in CD patients with ileal disease compared to patients with colonic localization; in contrast, in our study, the ileal localization was significantly associated with increased serum TBARS levels. We previously reported that disease located in the upper part of the intestine is a risk factor for recurrence when compared to diseases located in the distal ileum and colon [49].

The serum/plasma total antioxidant activity is the sum of the contribution of endogenous (uric acid, bilirubin, albumin) and exogenous (medications and food-derived) antioxidants, thus this disagreement may be due, not only to the complexity of the disease, but also to the variability in patient medications and dietary habits or supplement use. In this context, we noted differences in AOPP values associated with the treatment with mesalazine, known to exert anti-inflammatory and antioxidant effects, and in the FRAP levels that were significantly higher in patients under azathioprine treatment, which may be related to its contribution to oxidative stress [50].

Moreover, the lack of an association between circulating markers of oxidative damage and total antioxidant capacity suggests that in CD, oxidative stress is not just the result of an imbalance between oxidants and antioxidants, but may prime, at least in the acute phase of the disease, pro-inflammatory mechanisms through RAGE activation.

The main limitations of this study are the relatively small sample size, the cross-sectional design, the lack of follow-up, and of recurrent measurements during the course of the disease, which are necessary to validate the relevance of using oxidative stress markers in the clinical setting.

## 5. Conclusions

Despite these limitations, our data provide evidence that circulating AOPP and TBARS levels are significantly elevated in CD patients with severe relapse, suggesting that these parameters could be evaluated in a prospective, larger study on the progression of CD disease, as biomarkers for diagnosis or monitoring of CD patients. Moreover, our results indicate that AOPP/AGEs activation of RAGE signaling should be explored for diagnostic or therapeutic purposes in immune-mediated diseases such as CD.

## Figures and Tables

**Table 1:** Demographic and clinical characteristics of Crohn’s patients and healthy volunteers enrolled in the study.

Demographic and Clinical Characteristics	Crohn’s Patients	Controls
*n*	*54*	*17*
**Gender**		
-male	28 (51.9%)	7 (41.2%)
-female	26 (48.1%)	10 (58.8%)
**Age** (years)	42.12 ± 2.055	42.41 ± 3.73
**Disease duration** (years)	12.86 ± 1.37	
**Diagnostic delay** (months)	78.89 ± 16.32	
**Smoke habit**		
no	19 (38.0%)	8 (47.1%)
yes	16 (32.0%)	5 (29.4%)
former	15 (30.0%)	4 (23.5%)
**CDAI**	233.6 ± 5.66	
**Disease location**		
-Ileum	28 (53.8%)	
-Colon	18 (34.6%)	
-Ileo-colon	6 (11.5%)	
**Disease behavior**		
-Inflammatory	4 (7.5%)	
-Stricturing	28 (52.8%)	
-Fistulizing	3 (5.7%)	
-Stricturing and Fistulizing	18 (34.0%)	
**Extra-intestinal disease**		
-Skin	7/46 (15.21%)	
-Arthritis	12/46 (26.1%)	
**Perianal disease** yes/no	23/28 (45.10%)	
**Recurrence**		
-yes	30 (57.69%)	
-no	22 (42.31%)	
**Multiple operations**		
1	22 (42.31%)	
2	15 (28.85%)	
3	15 (28.85%)	
**Allergies** yes/no	19/25 (43.2%)	
**Familial IBD** yes/no	15/34 (30.6%)	

CDAI = Crohn disease activity index. Data are means ± SE or absolute and relative frequencies.

**Table 2:** Mean values of oxidative markers in serum samples from Crohn’s patients or healthy volunteers.

Oxidative Markers	Crohn’s Patients	Controls	
*n*	*54*	*17*	
FRAP, µM Fe^2+^	368.2 ± 90.72	343.8 ± 100.30	NS
TBARS, µM	4.00 ± 1.35	3.11 ± 0.45	*p* < 0.01
AGEs, AU	293.3 ± 108.80	216.0 ± 35.03	*p* < 0.01
AOPP, µmol/g of proteins	11.25 (5.02–15.15)	1.36 (0.75–2.70)	*p* < 0.0001
CO, nmol/mg of proteins	0.122 (0.095–0.146)	0.074 (0.061–0.12)	*p* < 0.01

Means ± SD or median (interquartile range 25–75); *p*-values by t test or by Mann–Whitney test. AU: Arbitrary Units.

**Table 3:** Multiple regression analysis of factors associated with circulating TBARS in Crohn’s patients.

Parameter	Estimate Coefficient	*p*-Value
CONSTANT	1.89611	0.0113
**Disease Location**	−0.63005	0.0185
**First Clinical Presentation**	0.270233	0.0089
**Smoking**	0.865863	0.0005
**Disease Behavior**	0.383783	0.0275
**Allergies**	0.997169	0.0001

**Table 4:** Multiple regression analysis of factors associated with the oxidative score in Crohn’s patients.

Parameter	Estimate Coefficient	*p*-Value
CONSTANT	0.490099	0.0159
**Smoking**	0.420792	0.0113
**Skin Extension**	0.782178	0.0245

## Supplementary Materials

The following are available online at https://www.mdpi.com/2076-3921/8/9/378/s1. Table S1: Correlations among markers of oxidative stress in Crohn’s patients, Table S2: Correlations among markers of oxidative stress in healthy volunteers.
