# Supplementary material for: Analysis of Oxidative Stress-Related Markers in Crohn’s Disease Patients at Surgery and Correlations with Clinical Findings
Source: Antioxidants (Basel). 2019 Sep 6;8(9):378. doi: 10.3390/antiox8090378 (PMC6771139; doi:10.3390/antiox8090378)
Supplement: Supplementary file 1 [file antioxidants-08-00378-s001.pdf]

antioxidants

MDPI

Supplementary Table 1. Correlations among markers of oxidative stress in Crohn's patients.

	FRAP	CO	TBARS	AGEs	AOPP
**FRAP**		0.2477	0.4830	0.2553	0.2863
		(39)	(39)	(39)	(39)
		0.1284	0.0018	0.1167	0.0773
**CO**	0.2477		0.4100	0.2849	0.4789
	(39)		(39)	(39)	(39)
	0.1284		0.0095	0.0788	0.0020
**TBARS**	0.4830	0.4100		0.3352	0.5298
	(39)	(39)		(39)	(39)
	0.0018	0.0095		0.0370	0.0005
**AGEs**	0.2553	0.2849	0.3352		0.1256
	(39)	(39)	(39)		(39)
	0.1167	0.0788	0.0370		0.4462
**AOPP**	0.2863	0.4789	0.5298	0.1256	
	(39)	(39)	(39)	(39)	
	0.0773	0.0020	0.0005	0.4462	

Data reported in table are correlation coefficients, (sample size: number of complete data) and p-values.

Supplementary Table 2. Correlations among markers of oxidative stress in healthy volunteers.

	FRAP	CO	TBARS	AOPP	AGEs
FRAP		0.5149	0.1760	0.3106	0.1350
		(16)	(16)	(16)	(16)
		0.0413	0.5144	0.2417	0.6180
CO	0.5149		0.3851	0.5335	0.1092
	(16)		(16)	(16)	(16)
	0.0413		0.1407	0.0333	0.6873
TBARS	0.1760	0.3851		0.6053	0.2402
	(16)	(16)		(16)	(16)
	0.5144	0.1407		0.0130	0.3703
AOPP	0.3106	0.5335	0.6053		-0.2030
	(16)	(16)	(16)		(16)
	0.2417	0.0333	0.0130		0.4508
AGEs	0.1350	0.1092	0.2402	-0.2030	
	(16)	(16)	(16)	(16)	
	0.6180	0.6873	0.3703	0.4508	

Data reported in table are correlation coefficients, (sample size: number of complete data) and p-values.
